# Mechanochemical feedback loops in contact-dependent fate patterning

**DOI:** 10.1016/j.coisb.2023.100445

**Published:** 2023-03

**Authors:** T. Dullweber, A. Erzberger

**Affiliations:** 1 Cell Biology and Biophysics Unit, European Molecular Biology Laboratory, Meyerhofstraße 1, Heidelberg, 69117, Germany; 2 Department of Physics and Astronomy, Heidelberg University, Heidelberg, 69120, Germany

**Keywords:** Symmetry breaking, Notch, Self-organization, Tissue mechanics, Adhesion

## Abstract

To reliably form and maintain structures with specific functions, many multicellular systems evolved to leverage the interplay between biochemical signaling, mechanics, and morphology.

We review mechanochemical feedback loops in cases where cell–cell contact-based Notch signaling drives fate decisions, and the corresponding differentiation process leads to contact remodeling. We compare different mechanisms for initial symmetry breaking and subsequent pattern refinement, as well as discuss how patterning outcomes depend on the relationship between biochemical and mechanical timescales.

We conclude with an overview of new approaches, including the study of synthetic circuits, and give an outlook on future experimental and theoretical developments toward dissecting and harnessing mechanochemical feedback.

## Highlights

• The mechanics and geometry of cell–cell contacts affect Notch signaling interactions.
• Mechanochemical feedback loops arise, when Notch, in turn, regulates contact remodeling.
• Contact-dependent mechanochemical feedback coordinates fate patterning in diverse contexts.

## Graphical abstract

**Figure:**
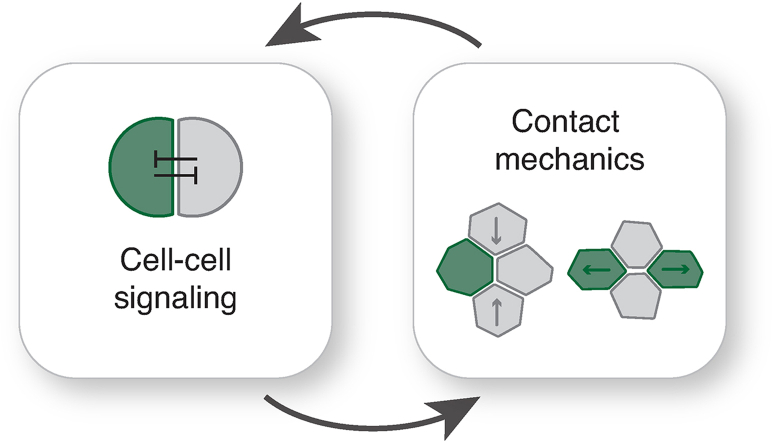

## Introduction

Multicellular processes, including development, regeneration, and homeostasis, require the precise spatial and temporal organization of a combination of mechanical, biochemical, and genetic factors [1,2,60]. Morphological or mechanical changes can impact biochemical signaling and vice versa, leading to mechanochemical feedback effects that can stabilize or alter the resulting structures [3,4]. For example, a change in the spatial distribution of receptors on a cell's surface [5] or the size of a lumen carrying ligands [6] can drastically alter where signals are transmitted, thereby determining morphogenetic outcomes.

**Figure 1:**
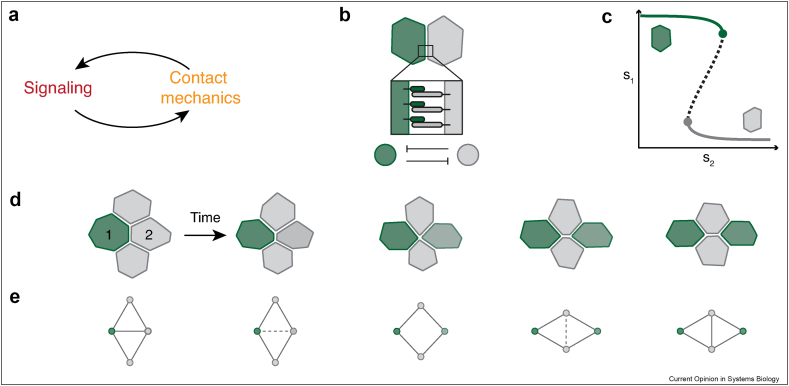
Contact-dependent signaling and mechanochemical feedback. (**a**) Cell–cell signaling upstream or downstream of adhesive or cytoskeletal components that control cellular contact area gives rise to mechanochemical coupling. (**b**) In many contexts, the activation of Notch receptors (gray) through ligands (green) on the surface of neighboring cells inhibits the expression of ligand molecules (lateral inhibition). These mutually inhibitory interactions amplify small differences in signaling state between interacting cells. The symmetry breaking into sending (green) and receiving (gray) cells is often accompanied by differentiation into distinct cell types. (**c**) A schematic bifurcation diagram for the signaling state of a cell *s*_1_ as a function of the state of the signaling partner *s*_2_ shows the sending state (green line) and the receiving state (gray line) separated by an unstable state (dashed line) [19, 23••]. Bistable signaling dynamics arise because the ability of a cell to send a signal is suppressed upon receiving a signal. (**d**) Schematic representations show a four-cell T1 transition in which a sending cell 1 loses contact with receiving cell 2, which consequently becomes a sending cell. In *Drosophila* cone cells, such a topological transition occurs downstream of Notch, thereby coupling the timing of the transition to the signaling dynamics [14•]. (**e**) Network schematics show the cell–cell contact changes during the topological transition. In principle, a direct dependence of contact remodeling on the concomitant changes in the exchanged signals constitutes a means for cells to sense and adapt to the progression of a topological transition. (For interpretation of the references to color in this figure legend, the reader is referred to the Web version of this article.)

When signaling interactions are restricted to direct cell–cell interfaces, feedback may arise naturally between cellular mechanics, morphology, and signaling (Figure 1a). Signaling pathways, including Notch [7], PCP [8], and Eph/Ephrin [9], rely on direct interactions between membrane-bound receptor and ligand molecules, such that the geometry of cell–cell contacts in a given tissue determines the possible signaling partners and affects their interactions. Notch signaling states, for example, correlate with the area of cell–cell contacts in different *in vivo* contexts [10], as well as *in vitro* where cell–cell configurations can be controlled by micropatterned bowtie-shaped wells [11]. Reaction-diffusion modeling can link the area dependence to the diffusion characteristics of receptors and ligands [12], and simulations confirm that the size and duration of contacts can impact signaling outcomes [13]. Contact-based signals can induce responses which, in turn, affect cell–cell contacts [9,14•]. For example, contact-based signaling often regulates cell fate and differentiation [15]. Over the course of differentiation, cells change their gene expression profile, morphology, and mechanical properties to acquire the features corresponding to their respective target cell type. These processes may concomitantly remodel the cell–cell contacts through which the differentiation-inducing signals are exchanged [16]. Changes in cell–cell adhesion or the formation of cytoskeletal protrusions alter cellular contacts and contact-dependent signaling interactions. Indeed, cells exchange contact-dependent signals over varying distances through protrusions of diverse sizes and shapes [17,18]. Bristle patterning in *Drosophila*, for example, is thought to involve Notch interactions through extended basal protrusions [19,20]. Moreover, transient signaling protrusions regulate both the timing and location of neuronal differentiation in the developing zebrafish spinal cord [21]. Similarly, changes to the topology of the cell–cell contact network—for example, due to cell division [22,23••], rearrangements [14•], or cell delamination from an epithelial monolayer [24•]—can affect fate patterns by initiating or terminating the exchange of signals or by changing the number of signal-sending cells [25•,^26^].

We review how mechanochemical feedback coordinates contact-dependent fate patterning in different *in vivo*, *ex vivo,* and synthetic contexts. We focus on examples that involve mutually inhibitory signaling interactions mediated by the Notch pathway or related synthetic circuits that lead to divergent cell fates (Figure 1b–c) [27,15]. We discuss contact-geometry-dependent feedback rather than effects arising from any direct mechanosensitivity of the Notch pathway [28].

In the context of fate patterning, symmetry breaking describes a process in which two or more initially similar cells acquire different properties, for example, through the amplification of small differences in biochemical composition or from a mechanical instability. We first cover cases in which the initial symmetry breaking occurs via signaling interactions with subsequent mechanical changes and spatial rearrangements that refine and stabilize the resulting spatial pattern of fates. Then, we consider how signaling modulates a pattern arising from a preceding mechanical symmetry breaking, followed by an example with multiple interlinked signaling and contact remodeling steps. Finally, we discuss how synthetic signaling circuits may help decipher mechanochemical feedback loops and conclude with an outlook on future directions.

## Signaling guides contact dynamics: fate patterning and adhesion

When the adhesive and cytoskeletal machineries governing cellular shape changes respond dynamically to differentiating signals, feedback effects can arise between signaling and the dynamics of cellular contacts. In the following, we discuss cases in which such contact-based mechanochemical feedback enables the coordination of signaling-dependent cellular processes with the dynamic spatial arrangement of the cells.

For example, Notch signaling regulates the intercalation of cone cells in the developing *Drosophila* eye [14•]. These cells are engaged in Notch signaling while undergoing a slow T1 transition over approximately 10h, in which the contact area between a signal-sending and a signal-receiving cell decreases and is eventually lost (Figure 1d–e). Blocking transcription downstream of Notch leads to intercalation defects, suggesting that the process is governed by feedback between contact remodeling and signaling, likely through heterotypic adhesion between the diverging cell types.

In vertebrates, Notch-dependent lateral inhibition drives the fate patterning of mechanosensory epithelia [29]. In these organs, the constituting cells acquire a sensory fate or a supporting cell fate according to the outcome of mutually inhibitory Notch signaling interactions [29, 30, 31], and the two cell types acquire a precise mosaic organization in which each sensory cell is surrounded by non-sensory supporting cells. There are distinct differences in the cellular contact dynamics during fate patterning across mechanosensory epithelia from different organs and species [23••,^32^,33••,^34^].

**Figure 2:**
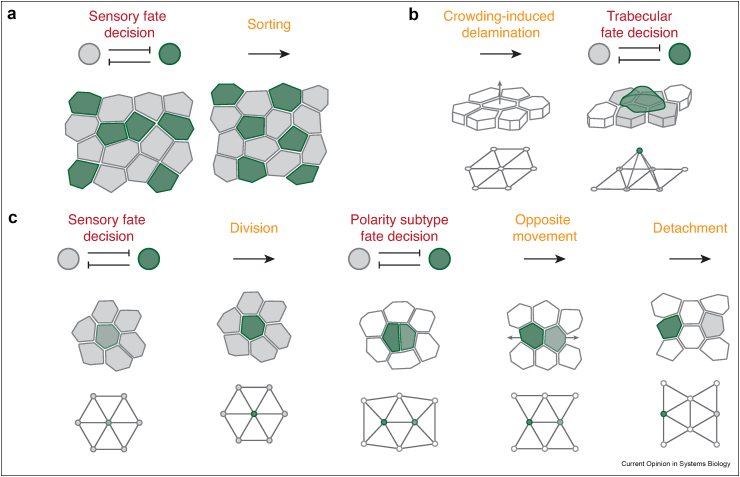
Mechanochemical fate patterning. (**a**) Notch-dependent fate determination followed by cellular rearrangements leads to precise mosaic arrangements of sensory cells (green) and supporting cells (gray) in mammalian mechanosensory epithelia [33••, 34]. (**b**) Mechanically induced cell–cell contact changes control Notch-dependent fate decisions in zebrafish heart development. The crowding-induced delamination of cells from the initially monolayered myocardial epithelium enables Notch-dependent lateral inhibition of delamination and trabecular fate in adjacent cells [24••]. (**c**) Notch-dependent cell–cell contact changes control distinct instances of signaling in zebrafish mechanosensory organs. Notch signaling specifies a sensory progenitor cell (green) among supporting cells (gray). This cell divides into two daughter cells that engage in a second instance of signaling to specify opposite polarity fates (green and gray), triggering oppositely oriented cell movements that terminate the signaling interactions by physically separating the cells [23••]. (For interpretation of the references to color in this figure legend, the reader is referred to the Web version of this article.)

The mammalian auditory organ develops from a post-mitotic prosensory epithelium. Therefore, cell divisions do not change the contact-network topology during signaling. Mechanical processes, including neighbor exchanges, affected by Notch-dependent changes in cellular properties, however, impact the final fate patterns, as *in vivo* and explant studies in mouse have shown [33••,^34^,^35^]. Live imaging of explants and vertex model simulations suggest that Notch signals create a salt-and-pepper distribution of sensory cells and supporting cells that express heterotypic adhesion molecules and acquire different mechanical properties, which—in interaction with tissue-level shear stresses in the developing organ—facilitate a mechanical sorting process that arranges sensory and supporting cells into a precise mosaic pattern (Figure 2a, [[33••], 34]).

## Contact dynamics guide signaling: form and fate in heart organogenesis

In some contexts, mechanical processes—such as buckling [36] or contractile instabilities [37]—initiate fate patterning, and signaling subsequently stabilizes or further modifies the arising structures.

In the developing zebrafish heart, for example, proliferation-induced crowding of cells creates tension heterogeneities in the epithelium of the early myocardium [24••,^38^]. Cells with the highest actomyosin contractility delaminate and seed the formation of an inner layer, which later gives rise to the muscular structures of the trabeculae. At the onset of trabeculation, as the single cells delaminate, the neighboring cells activate Notch, while the delaminated cell remains in a Notch-negative sender state (Figure 2b). Interestingly, this contractility-induced delamination is sufficient to trigger Notch activation in the adjacent, non-delaminated cells. In turn, Notch signaling affects the actomyosin machinery in these cells and counteracts their delamination. Of note, Notch-positive cells have larger apical surfaces than the delaminating Notch-negative cells, indicating a role for the signaling contact area in triggering Notch activation [24••].

Rather than being temporally separated processes, mechanical rearrangements and signaling interactions occur on the same timescale during cardiac trabeculation, and their interplay regulates the spatial distribution of cell types. In the next section, we discuss how interlinked signaling and contact remodeling permits the coordination of distinct differentiation events within the same organ.

## Contact dynamics and signaling interlinked

In contrast to their mammalian counterparts (Section 1), mechanosensory epithelia in other vertebrates remain proliferative after patterning and, in some cases, exhibit remarkable regenerative capacity [39,40]. In the sensory organs of the zebrafish lateral line, for example, live imaging shows high rates of proliferation with cells rearranging and continuously reshaping the contact-network topology throughout development and regeneration [23••,^31^]. How are signaling and contact remodeling coordinated under such circumstances? Indeed, the Notch pathway regulates two interlinked fate decisions in these organs that are timed by Notch-dependent contact dynamics (Figure 2c). In the lateral line, two subtypes of sensory cells arise in pairs from the divisions of progenitor cells [41,42]. One instance of Notch signaling specifies the sensory progenitor cells among the supporting cells [31,43], while a second instance breaks the symmetry between the daughter cells to produce one of each sensory subtype [23••,^44^,^45^]. Subsequently, the two cells form oppositely oriented actin protrusions and move away from one another, while supporting cells intercalate, possibly aided by heterotypic adhesion [23••]. The two steps are coordinated by the differentiation-induced changes in the topology of cell–cell contacts. First, the progenitor division creates a new interface between the daughter cells through which they engage in lateral inhibition, and then the elicited fate maturation process terminates this contact in a self-coordinated fashion. Thus, the formation and elimination of physical contacts between cells keep the different functions of the same pathway spatiotemporally separate. It will be interesting to explore whether the mechanochemical self-organization of fate decisions in this dynamically rearranging organ facilitates its capacity for regeneration.

## Dissecting feedback in synthetic systems

Within a dynamic environment *in vivo*, cells receive a range of molecular signals that influence each other and are affected by ongoing external processes. It is challenging, for example, to decouple feedback effects between contact-dependent signaling and contact remodeling from the cross-talk with other signaling pathways [46]. Engineering signaling receptors to construct circuit motifs with desired properties facilitates isolating mechanisms of interest and testing predictions [47,48].

This approach can link contact-mediated signaling to downstream effectors of cellular mechanics directly, that is, independent of other molecular pathways. Synthetic Notch receptors permit the design of custom input and output domains that can be used to program contact-dependent transcription [49,50]. Indeed, engineering cells in which receptor activation suppresses the expression of the corresponding ligand leads to mutual inhibition that breaks the symmetry and bifurcates cells into two groups of either high or low ligand expression. Moreover, programming the circuits to induce the expression of homophilic adhesion molecules downstream of receptor activation gives rise to the formation of compact structures with a core of receiving cells surrounded by ligand-expressing sending cells [50]. The results demonstrate that a simple feedback between contact-based signaling and contact remodeling can break the symmetry among initially uniform cells and drive spatial organization into distinct layers.

### Outlook

In multicellular systems where cells exchange signals through direct physical contacts, signaling is affected by cellular rearrangements due to proliferation, migration, or cell shape changes. When signaling directly influences cell–cell contact mechanics, feedback effects can arise, which are harnessed in diverse ways toward generating spatial structures or dynamical states during development, regeneration, and homeostasis [23••, 24••, 25•].

Gaining a formal and predictive understanding of mechanochemical systems is a core goal of theoretical biological physics. It motivates novel combinations of theoretical approaches from cellular biophysics, the collective dynamics of active matter, and the nonlinear dynamics of cell–cell communication [3,19,23••,^51^]. For example, recent work on communicating active matter outlines how modeling collective motion with signaling dynamics reveals new principles of multicellular organization [52]. New technologies, especially in the field of live imaging combined with optogenetics and the development of novel *ex vivo* and *in vitro* platforms, allow us to monitor and manipulate the spatial dynamics of cells and their signaling states in unprecedented ways [47,53,54]. Increasingly complemented by theoretical approaches [13,34,55, 56, 57, 58, 59], these advances will reveal patterning regimes and self-organizing motifs specific to mechanochemical systems and improve the targeted manipulation and reconstitution of dynamical multicellular structures.

## Conflict of interest statement

Nothing declared.

## Data Availability

No data were used for the research described in the article.
